# Socio-demographic parameters and non-cardiac comorbidity related to self-perceived quality of life in young adults after neonatal arterial switch operation for transposition of the great arteries

**DOI:** 10.1007/s00380-022-02188-8

**Published:** 2022-10-28

**Authors:** Hedwig H. Hövels-Gürich, Corinna Lebherz, Bettina Korte, Jaime F. Vazquez-Jimenez, Nikolaus Marx, Gunter Kerst, Michael Frick

**Affiliations:** 1 Department of Pediatric Cardiology, University Hospital RWTH Aachen, Pauwelsstr. 30, 52074 Aachen, Germany; 2 Department of Cardiology, University Hospital RWTH Aachen, Aachen, Germany; 3 Department of Cardiac Surgery for Congenital Heart Defects, University Hospital RWTH Aachen, Aachen, Germany; 4 Superregional Center for Adults with Congenital Heart Disease, University Hospital RWTH Aachen, Aachen, Germany

**Keywords:** Adults with congenital heart disease, Transposition of the great arteries, Arterial switch operation, Health-related quality of life, Non-cardiac comorbidity, Socio-demographic parameters

## Abstract

Evaluating the relation of non-cardiac comorbidity and socio-demographic factors to physical and mental health-related quality of life (QOL) which has been partially found at elevated risk in young adults after neonatal arterial switch operation (ASO) for transposition of the great arteries (TGA). In a prospective reassessment study, results of 92 unselected young adults (22.8 ± 2.6 years) having undergone evaluation of QOL (SF-36) were related to non-cardiac comorbidity with special respect to neurologic and psychiatric comorbidity and to socio-demographic parameters. Neurologic (14%) contrary to psychiatric comorbidities (6.5%) were more frequent than in the general population. The educational level was higher, the rate of unemployment was double as high compared to the average German population. Significant inverse relations (*p* = 0.006 to 0.033) existed between physical health domains (physical functioning and general health perception) and non-cardiac, neurologic, and psychiatric comorbidity, as well as correlations between the latter domains and socio-economic status, educational level, and worse employment status (Spearman 0.22–0.41, *p* < 0.0001 to 0.036). Mental health domains (vitality, social functioning, psychical health) were significantly inversely related with neurologic and psychiatric comorbidity (*p* = 0.002 to 0.048) and correlated with higher educational level (Spearman 0.25, *p* = 0.019). Neurologic and psychiatric comorbidities and socio-demographic parameters are significant risk factors for a reduced QOL concerning physical and mental health in young adults with TGA after ASO. Standardized QOL measurement should be part of routine screening programs to detect subclinical physical, neurodevelopmental, and psychosocial comorbidity.

**Graphical abstract:**

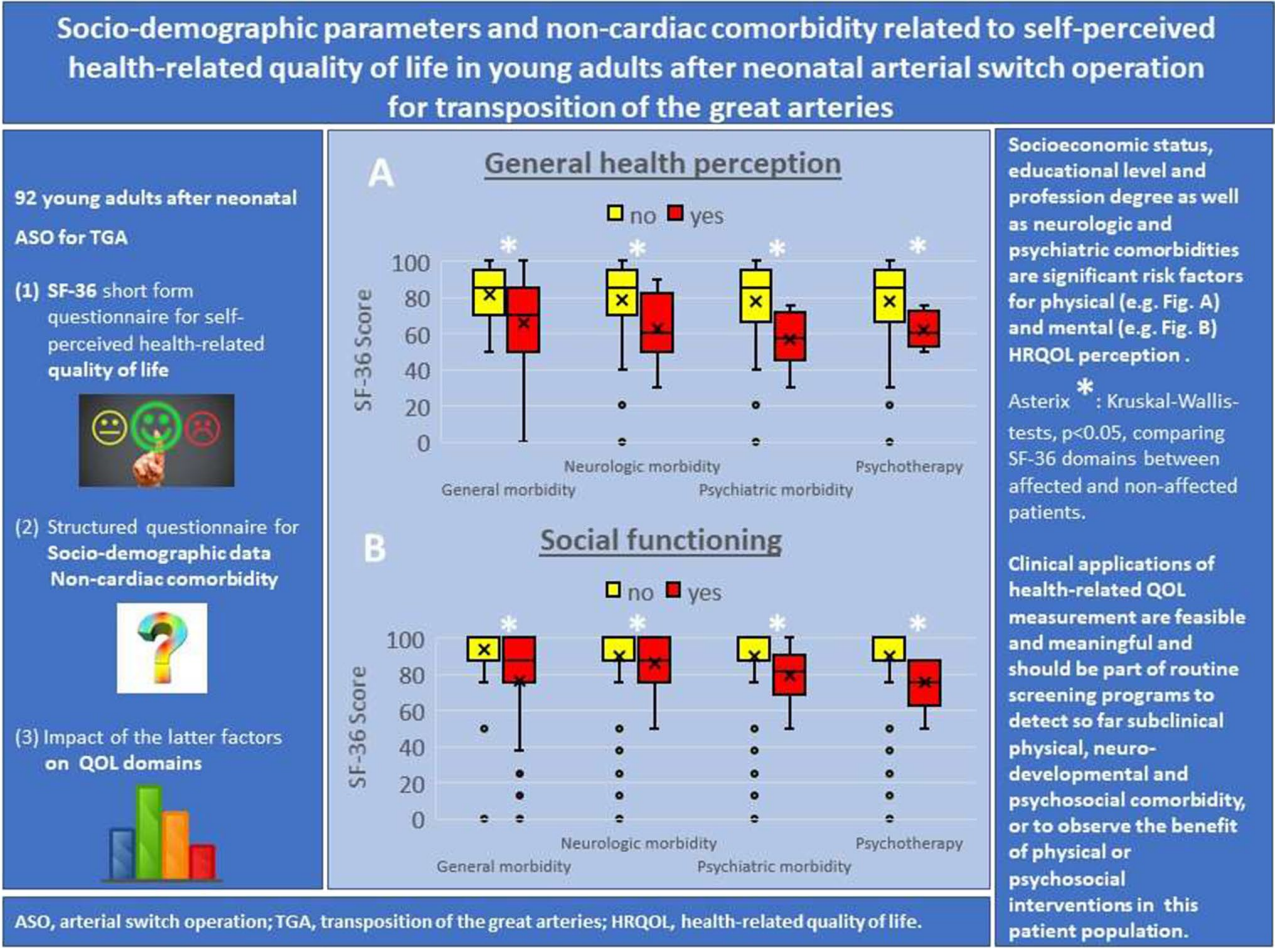

## Introduction

With a prevalence of almost 1% of live births, congenital heart defects (CHD) represent the most common isolated human malformation. Simple transposition of the great arteries (TGA) is the most common cyanotic heart defect in newborns, accounting for around 3–5% of congenital heart defects. Untreated, TGA results in very poor short-term survival rates. Therefore, the arterial switch operation (ASO) in newborns has been established as the surgical method of choice for early anatomical correction since the late 1980s. The aorta and pulmonary artery are swapped proximally, and the coronary arteries are reimplanted. The surgical method has a high long-term survival rate of over 90% and promises an almost normal physical performance with an overall good long-term course on the part of the heart [1]. A good 30 years after the world's first operations of this type, there is a growing number of young adults whose long-term quality of life (QOL) is the focus of interest on an equal level with the cardiac functional status [2, 3]. However, despite good long-term cardiologic results, an increased incidence of cognitive, neuropsychologic and psychiatric issues has emerged in this patient group [4–6] influencing the psychosocial domains of their self-rated health-related quality of life (HRQOL). Partly independent of the severity of the heart defect, both physical and psychosocial morbidity can significantly influence QOL in adult patients with congenital heart disease (ACHD) [7–11].

In the context of our institution’s extensive ASO reassessment program since 1997 [12–14], we have recently shown, that despite maintained, normal to low normal average outcomes for self-rated physical and mental QOL, an increased proportion of this cohort of young adults is at significantly elevated risk for QOL difficulties and impairments (scores below the first and second negative standard deviation, resp.), especially with respect to social and emotional functioning as well as to physical functioning and general health perception. Subjective (New York heart association (NYHA) functional class) and objective (cardiopulmonary exercise test capacity) markers of heart failure have recently been evaluated as significant risk factors [15]. The present study aimed to assess associations between our patients’ self-perceived QOL and socio-demographic as well as non-cardiac comorbidity as possible additional risk factors.

## Methods

### Demographic, surgical and cardiologic follow-up data

This monocentric prospective study consisted of 92 unselected young adults who had undergone ASO for simple TGA as neonates, aged 18–29 (mean 23) years at reevaluation. The surgical procedure of neonatal ASO was performed as described previously [12]. Surgical and cardiologic follow-up data have been recently reported [15]. At the time of reassessment, 7 patients. (7.6%) had undergone a total of 8 cardiac reoperations during childhood or adolescence, and 10 pts. (10.9%) a total of 15 catheter interventions. 4 patients (4.3%) were on cardiovascular medication (beta blocker, afterload-lowering). 88% of the patients were in NYHA class I without limitation, 12% in class II with mild limitation in daily life. The evaluation was performed prior to the Corona pandemic.

The study was designed as a case series with published controls and prognostic factor analyses. The study protocol conforms to the ethical guidelines of the 1975 Declaration of Helsinki as reflected in a priori approval by the local Ethical Medical Committee (Medical Faculty RWTH Aachen university, Nr. EK 243/14). Written informed consent was obtained from each patient.

### Socio-demographic factors

Socio-demographic factors like socio-economic status [16, 17], educational level, profession degree including employment status as well as family status were collected by a standardized questionnaire and comparisons to the current German population were made, where available (Table 1).

**Table 1:** Socio-demographic parameters and non-cardiac comorbidity in 92 young adults after neonatal ASO for TGA

Variable	Definition	Results	German population
Socio-economic status	Social class according to socio-economic situation [16]:		
	Upper/upper middle class	32.6%	15%^a^
	Middle class	57.6%	61%
	Subclass/worker	9.8%	24%
Educational level	High school (qualifying for university admission)	44.6%	27.3%^b^
	Secondary school	32.6%	29.0%
	Elementary school	19.6%	35.6%
	Attending school		4.3%
	No final school examination	3.3%	3.8%
Profession degree/employment status	According to requirement level:		
	High	34.8%	26.9%^c^
	Middle	22.8%	57.0%
	Basic	31.5%	10.5%
	No employment	10.9%	5.6%
Family status	Living in partnership	46%	ca. 50%^d^
	Pregnancy (≥ 1)	9.4%	
	Offspring (≥ 1)	6.7%	ca. 20%
Non-cardiac comorbidity	General morbidity in total	30.4%	
	Neurologic morbidity^e,g^	14.1%	
	Psychiatric morbidity^f,h^	6.5%	
	Other^i^	9.8%	
Non-cardiac therapy	Medication in summary	15.2%	
	Psychiatric medication	5.4%	
	Other medication^j^	9.8%	
	Psychotherapy	5.4%	

^a^Bünning M. (2018). Soziale Lagen und soziale Schichtung, p 260–261. In: Sozialstruktur und soziale Lagen; Auszug aus dem Datenreport 2018. Wissenschaftszentrum Berlin für Sozialforschung GmbH, Berlin, Germany. Retrieved February 9, 2022, from https://www.destatis.de/DE/Service/Statistik-Campus/Datenreport/Downloads/datenreport-2018-kap-7.pdf?_blob=publicationFile

^b^Kurz und knapp (2014). Bildungsstand der Bevölkerung. Bundeszentrale für politische Bildung. Bundeszentrale für politische Bildung, Bonn, Germany. Retrieved February 9, 2022, from https://www.bpb.de/kurz-knapp/zahlen-und-fakten/soziale-situation-in-deutschland/61656/bildungsstand-der-bevoelkerung/

^c^Arbeitslosenquote in Deutschland 2017. Statista, Germany. Retrieved February 9, 2022, from https://de.statista.com/infografik/8338/deutschlands-arbeitslosenquote/ Beschäftigung in Deutschland nach Anforderungsniveau 2017. Bundesagentur für Arbeit, Nürnberg, Germany. Retrieved February 9, 2022, from https://statistik.arbeitsagentur.de/DE/Navigation/Statistiken/Interaktive-Statistiken/Berufe-auf-einen-Blick/Berufe-auf-einen-Blick-Anwendung-Nav.htm

^d^Wippermann C. (2014). Kinderlose Frauen und Männer. Bundesministerium für Familie, Senioren, Frauen und Jugend, Germany. Retrieved February 9, 2022, from https://www.bmfsfj.de/resource/blob/94130/bc0479bf5f54e5d798720b32f9987bf2/kinderlose-frauen-und-maenner-ungewollte-oder-gewollte-kinderlosigkeit-im-lebenslauf-und-nutzung-von-unterstuetzungsangeboten-studie-data.pdf Geburten in Deutschland (2021). Statistisches Bundesamt, Wiesbaden, Germany. Retrieved February 9, 2022, from https://www.destatis.de/DE/Themen/Gesellschaft-Umwelt/Bevoelkerung/Geburten/_inhalt.html

^e^Incidence of total neurologic morbidity in the European population (2015): 1.54%. Retrieved November 11, 2021, from https://de.statista.com/statistik/daten/studie/180618/umfrage/anzahl-neurologischer-erkrankungen

^f^Incidence of psychiatric morbidity in Germany at age 20–29 years (2011): male 29.6%, female 37.3%. Retrieved November 11, 2021, from https://de.statista.com/statistik/daten/studie/221496/umfrage/psychische-erkrankungen-in-der-deutschen-allgemeinbevoelkerung

Patients’ incidences (%)

^g^5.4% fine motor/coordination dysfunction, 2.2% spastic hemi/tetraplegia, 2.2% epilepsy, 1.1% psychomotor retardation, 1.1% migraine, 1.1% tic dysfunction, 1.1% brain tumor

^h^2.2% executive dysfunction; each 1.1%: brain organic psychosyndrome, obsessive compulsive disorder, personality disorder, depression

^i^5.4% respiratory allergy, 4.4% hypothyroidism (under substitution), 1.1% myelodysplastic syndrome

^j^5.4% thyroxin, 2.2% antiepileptics, 1.1% pituitary substitution, 1.1% acyclovir

### Non-cardiac comorbidity

A standardized questionnaire assessed the incidence of non-cardiac comorbidity with special respect to neurologic and psychiatric morbidity, as well as non-cardiac therapy including medication and psychotherapy. Comparisons to the current German population were made, where available (Table 1).

### Quality of life results related to socio-demographic factors and non-cardiac comorbidity

Our recently reported results of standardized evaluation of QOL (SF-36) [15] as a generic measure of subjective health status [18] were related to socio-demographic and to non-cardiac comorbidity factors (Table 2) aiming at the detection of potential non-cardiac risk factors for a reduced QOL outcome.

**Table 2:** Correlations between health-related quality of life test results and socio-demographic parameters in 92 young adults after neonatal ASO

SF-36 scores	Socio-economic status (grade 0: lowest to 2: highest)		Educational level (grade 0: lowest to 3: highest)		Profession degree (grade 0: unemployment to 3: highest)	
	Spearman^a^	*p* value	Spearman	*p* value	Spearman	*p* value
Physical health						
Physical functioning	**0.33**	**0.002**	**0.22**	**0.036**	**0.41**	**< 0.0001**
Physical role function	0.01	0.909	0.07	0.584	0.12	0.252
Bodily pain	-0.11	0.322	− 0.05	0.515	0.09	0.374
General health perception	0.01	0.916	0.08	0.475	0.17	0.106
Mental health						
Vitality	− 0.07	0.525	− 0.03	0.820	− 0.04	0.710
Social functioning	0.15	0.169	0.08	0.432	0.15	0.163
Emotional role function	− 0.19	0.068	**0.25**	**0.019**	− 0.14	0.187
Psychical health	− 0.08	0.466	− 0.07	0.606	0.01	0.961

Significant correlations (*p* value < 0.05) in bold letters

^a^Spearman correlation coefficient

### Statistical analysis

Results were expressed by mean values and standard deviation (m ± SD), median and interquartile ranges, minimal and maximal values, or as percentages.

Chi-squared tests were used to test for differences between frequencies (socioeconomic status classes: patients vs norm [16, 17], representing an ordinal scale).

Univariate correlation analyses (Spearman correlation coefficients) were applied to analyze continuous variables to study the impact of influencing factors from socioeconomic status on SF-36 outcome parameters.

The significance of discrete non-cardiac comorbidity status variables on SF-36 outcome parameters was calculated by means of Kruskal–Wallis tests and presented as box plots.

For additional multivariable analyses, outcome parameters of the SF-36 dimensions were modeled using generalized linear models with a logit link function. Outcomes were scaled to range between 0 and 1. For each dimension score separate models were built using: (1) predictors of socioeconomic parameters, (2) non-cardiac comorbidity status parameters. Models were fitted using Maximum Likelihood estimation.

Statistical analysis was performed with the SPSS for Windows software, version 26 (SPSS GmbH Software, München, Germany) and R version 3.5.2 (R Core Team (2018). R: A language and environment for statistical computing. R Foundation for Statistical Computing, Vienna, Austria. URL https://www.R-project.org/). All statistical tests were performed at a significance level of 0.05.

## Results

### Socio-demographic factors (Table 1)

Socio-economic status of the cohort was different from the current normal German population with a markedly higher percentage of members in the upper middle class and a lowered percentage in the subclass. In this connection, the method of the subjective stratification of the social class is considered largely determined by the factual socio-economic status or social situation.

With respect to educational level, percentages of qualified degrees were higher than those of the average German population. In addition, the percentage of high profession degrees exceeded those of the normal population. Nevertheless, the rate of unemployment was almost double as high.

Family status in terms of living in partnership was like the normal population, whereas the offspring rate was found reduced to about one third of the average German young adult male or female population (reference sources in Table 1, footnotes).

### Non-cardiac comorbidity (Table 1)

About one third of the patients suffered from concomitant non-cardiac comorbidity, in which neurologic morbidities such as coordination disorders, spastic hemi- or tetraplegia and epilepsy (14.1%) as well as psychiatric morbidities (6.5%) including executive dysfunctions (e.g., goal-directed behavior, cognitive flexibility, planning) were the most prevalent disease groups, followed by respiratory allergies and hypothyroidism.

Non-cardiac medication was applied in a total of 15% in which psychiatric medication was prevalent in 5.4%. Moreover, 5.4% received psychotherapy. Overall, the incidence of neurologic comorbidity in our patient group exceeded the one expected for an age-appropriate average population, whereas the incidence of psychiatric comorbidity and therapy did not (reference sources in Table 1, footnotes).

### Univariate associations between outcome parameters

Spearman correlation analyses were calculated between the outcome parameters of the 8 SF-36 dimensions and 3 possible influencing continuous variables concerning socio-demographic parameters. Correlations (Spearman 0.22–0.41, *p* < 0.05; bold letters) are shown in Table 2.

With respect to the physical domains of SF-36, *physical functioning* was significantly correlated with better socioeconomic status, educational level and profession degree.

With respect to the mental domains of SF-36, *emotional role function* was significantly correlated with the educational level.

**Fig. 1:**
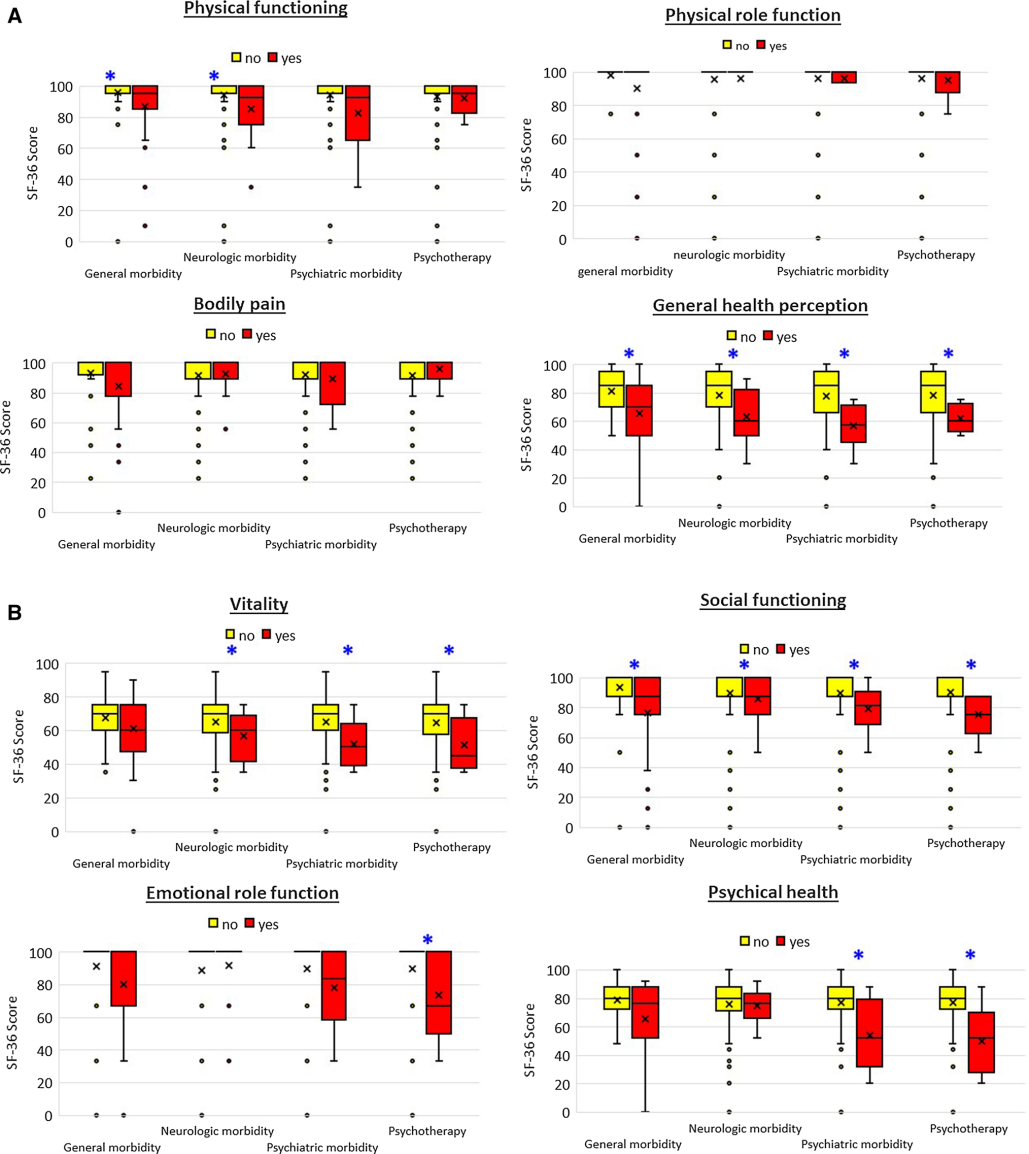
**a**, **b** Quality of life scores (SF-36) related to non-cardiac comorbidity status in 92 young adults after neonatal ASO. **a** Physical health with 4 subscores: physical functioning, physical role function, bodily pain, general health perception. **b** Mental health with 4 subscores: vitality, social functioning, emotional role function, psychical health. Red columns: affected patients. Yellow columns: non-affected patients. Sf-36 scores expressed as box plots with mean value (*x*), median, 25th and 75th percentiles, minimum and maximum value. *Significant test results (*p* value < 0.05) from Kruskal–Wallis tests comparing SF-36 domain scores between affected and non-affected patients (compare Table 3)

Kruskal–Wallis tests were calculated comparing the results of the 8 SF-36 dimensions with 4 discrete variables of non-cardiac comorbidity (subgroups of patients with or without non-cardiac comorbidity in total, neurologic comorbidity, psychiatric comorbidity, psychotherapy, resp). *P* values are displayed in Table 3, as also visualized by box plots in Fig. 1a, b.

**Table 3:** Comparison between health-related quality of life test results and non-cardiac comorbidity status in 92 young adults after neonatal ASO: 2 subgroups with or without morbidity

SF-36 scores	General non-cardiac morbidity in total *P* value^a^	Neurologic morbidity *p* value	Psychiatric morbidity *p* value	Psychotherapy *P* value
Physical health				
Physical functioning	**0.012**	**0.033**	0.071	0.273
Physical role function	0.583	0.412	0.520	0.398
Bodily pain	0.138	0.944	0.728	0.635
General health perception	**0.017**	**0.016**	**0.006**	**0.026**
Mental health				
Vitality	0.300	**0.019**	**0.026**	**0.048**
Social functioning	**0.003**	**0.037**	**0.013**	**0.002**
Emotional role function	0.647	0.659	0.074	**0.030**
Psychical health	0.323	0.355	**0.018**	**0.017**

Significant test results (*p* value < 0.05) in bold letters

^a^*P* values from Kruskal–Wallis tests comparing SF-36 domain scores between patients with/without morbidity (compare Fig. 1a, b)

### Multivariable analyses of influencing factors on outcome parameters

Seven outcome parameters of the SF-36 dimensions (except physical role function, due to reduced data variability) were modeled using generalized linear models. For each dimension score separate models were built using: (1) predictors of socio-demographic parameters (from Table 2), and (2) non-cardiac morbidity status parameters (from Table 3, except psychiatric morbidity and psychotherapy, due to reduced data variability). As a result, none of the 14 built generalized linear models was able to reveal any independent association between the SF-36 dimensions’ scores and the influencing factors due to weak evidence and high standard errors (data not shown).

## Discussion

Adults with congenital heart disease, as a heterogeneous population with heart defects of simple, moderate or severe complexity, are at increased risk for physical, psychiatric, psychosocial and socioeconomic problems, potentially leading to a reduced quality of life, partly independent of the severity of their heart defect [10, 11, 19–21]. Contrary, young adult patients with transposition of the great arteries after neonatal arterial switch operation, represent a uniform subgroup with a congenital heart disease of moderate complexity, providing an overall good cardiologic condition. Within our prospective arterial switch reassessment program [12–14, 22–24], we had recently reported on an unselected cohort of TGA patients at mean age 23 (18–29) years: despite a normal to borderline objective exercise capacity and a good NYHA status, they had a persistent elevated risk for a reduction of their HRQOL [15]. Therefore, the aims of the present study were (1) to assess socio-demographic parameters and non-cardiac comorbidity compared to the normal population, and (2) to evaluate the relation of the latter factors to the self-perceived HRQOL dimensions in these patients.

### Socio-demographic factors

In our patient group, socio-economic status as a potential risk factor for quality of life [11] was markedly elevated compared with the normal German population (Table 1). Educational level was even better providing a markedly higher rate of high school degrees in comparison to the normal population. In addition, we found an increased prevalence of employment with a high profession degree. However, unemployment was almost twice the rate, compared to the normal German population and even higher than in a cross-sectional survey from the National Registry for Congenital Heart Defects in Germany [25]. Living in partnership was in the normal range compared to the German population, but the offspring rate was found low for both genders, in which pregnancy has been basically well tolerated with good maternal outcomes after ASO for TGA [26].

### Non-cardiac comorbidity

With respect to the non-cardiac comorbidity, chronic neurologic illness comprises about 50% of our patients with a general comorbidity and is markedly increased compared to the normal European population, whereas the incidence of reported psychiatric comorbidity is not found elevated compared to the normal German population (Table 1). However, summarized neurologic and psychiatric illness requiring treatment even includes two thirds of the total non-cardiac chronic comorbidity. Regarding our longitudinal comparison with data at mean age 17 years, the current incidence of neurologic morbidity has slightly increased due to acquired conditions such as epilepsy, migraine, tic dysfunction, brain tumor, but is still dominated by pre-existing motor coordination dysfunctions and spastic paralysis. Despite encouraging overall neurodevelopmental outcomes, a significant minority of adolescents had psycho-intellectual performances below the expected level in our former study [14]. Advanced findings with respect to reduced neuropsychological outcome and increased risk of psychiatric disorders have been published from the Boston Circulatory Arrest Study at age 16 years: impaired cognitive function and parental stress at younger age as well as executive dysfunction deficits were at risk to increase psychosocial and psychiatric morbidity in adolescents after ASO [27, 28]. Continuing these challenges into the young adult age, cognitive and psychologic difficulties as well as increased risk of psychiatric morbidity in terms of depression and anxiety have been confirmed in patients after neonatal ASO for TGA with a higher lifetime prevalence of depression compared to healthy controls (43 vs. 19%) and anxiety disorders (54 vs 33%) [4–6]. In contrast, in patients after the formerly performed atrial redirection surgery for TGA, the proven incidence of psychiatric disease has been reported with 20%, and in mixed cohorts of adults with congenital heart defects, the rate of psychiatric disorders reached about 30% [6, 19]. Finally, the influences of multiple patient-related and procedure-related risk factors, including results of structural and functional brain MRI investigations, on neurodevelopmental and psychosocial issues after cardiac surgery during infancy have become a matter of debate for many years now [29–33].

### Analysis of influencing factors on HRQOL

#### Physical health dimensions

The self-perceived physical and psychosocial wellbeing had been assessed prior to the Corona pandemic using the SF-36, one of the most common and widely used standardized health-related non-disease-specific quality of life tests with German norm values from 2011.

From the spectrum of physical health dimensions, physical functioning was the dimension that most frequently received diverse influences as well from all assessed socio-demographic parameters as from summarized non-cardiac comorbidity and neurologic morbidity. The impact on the more overriding dimension of general health perception, was influenced by factors of summarized non-cardiac comorbidity, neurologic and psychiatric morbidity.

Our results about the impact on self-perceived physical wellbeing confirm significant associations of socio-demographic factors (socioeconomic status, educational level, employment status) with physical QoL, compared to previous studies in mixed ACHD patients [10, 20, 25]. In general, disease severity is reported to have an impact on the physical domains of QoL [25, 34].

Less data have been published about associations between physical health and non-cardiac morbidity in TGA patients. In contrast to the recent study conducted by Kalfa and Kasmi et al. [4–6], we also included patients with non-cardiac comorbidity, especially those with neurologic and neurocognitive disorders potentially disturbing quality of life. Nevertheless, in the aforementioned study, the authors reported lower satisfaction in the physical health domain. As expected, our data show a significant impact not only of neurologic, but also of psychiatric morbidity on physical health perception. It has been reported that, despite significant resilience to known neurologic, neuropsychologic and academic deficits, increased rates of ACHD patients, including those after ASO for TGA, present with reduced psychosocial functioning and increased risk of psychiatric morbidity, beginning in childhood and continuing into adolescence [27, 28]. In young adulthood, cognitive and psychologic difficulties increase the risk of poorer quality of life [4–6].

#### Mental health dimensions

Within the dimensions of mental health, social functioning received negative impact from non-cardiac comorbidity, neurologic and psychiatric morbidity. Vitality was also influenced by neurologic and psychiatric morbidity. The emotional role function was negatively influenced in patients needing psychotherapy; it was positively influenced by a better educational level. The negative impact on the overriding dimension of psychical health was focused on psychiatric morbidity and psychotherapy.

Our results about the impact factors on self-perceived mental health highlight the importance of neurologic, neurodevelopmental, and psychosocial morbidity, as outlined previously [11, 35]. In the pediatric population with CHD, high complexity heart defects with psychosocial morbidity factors as parental stress, posttraumatic stress disorder or anxiety symptoms have been found associated with reduced psychosocial wellbeing. Other studies evaluated factors as low self-perception, need for any medication, or lower cognitive function [6, 13]. In young adult patients with TGA after ASO, psychiatric disorders, possibly partly as result of pre-existing cognitive or psychologic difficulties, have been evaluated as risk factors for reduced psychosocial quality of life [4]. Especially a markedly elevated presence of mood and anxiety disorders significantly influences social interactions, employability, and achievement [5, 6] and hereby decreases self-perceived psychosocial wellbeing. Furthermore, female gender, less social support [20], or socio-demographic factors as increasing age, partnership, education level and unemployment [25] have been found negatively associated with mental health perception.

#### Synopsis and outlook

In our patient group, we have shown that:

1. Better physical health perception is significantly correlated with better socio-economic status, better educational level, but with lower employment status.
2. Better physical and mental health perceptions are significantly correlated with the absence of non-cardiac comorbidity, especially with absent neurological or psychiatric comorbidity.
3. Better physical and mental health perceptions are correlated with better objective exercise capacity and NYHA status [15].
4. Physical and mental health perceptions—as key parameters for quality-of-life evaluation—are plausible indicators for the aforementioned risk factors, also pointed out in studies on mixed ACHD groups [10, 25].

Being aware of the increased risks due to non-cardiac comorbidity in ACHD patients:

1. Physical and psychosocial interventions are deemed necessary, focused on training the patients’ comprehension of the disease and providing coping strategies leading to a better resilience [36, 37].
2. To date, limited observational intervention studies to improve the physical or psychosocial status of children and adults with CHD and their families have been successfully performed. The shift to systematic interventional research studies to improve QOL for children and adults with CHD has recently been designed, but efficient generalizable results are still lacking [38–40].
3. Physical and psychosocial interventions can be mandatory for individual patients’ benefit as well as for the general benefit of society, even in this population with a generally good cardiologic health [11, 20, 41–43]. Serial quality of life evaluations can be a useful measure to monitor the benefit of such interventions [6, 36, 37].

## Conclusion

Based on the fact that despite good results on average, an increased proportion of young adults with TGA after ASO is at elevated risk for QOL difficulties and impairments, especially with respect to physical, social and emotional functioning [15], potential non-cardiac risk factors have been assessed. In this study, different socio-demographic parameters as socio-economic status, educational level and employment status, and non-cardiac, especially neurologic and psychiatric comorbidities have been found as significant risk factors for physical as well as for mental health perception. Clinical applications of health-related quality of life measurement are feasible and meaningful and should be part of routine screening programs even in ACHD patients with a good cardiologic condition to detect so far subclinical physical, neurodevelopmental and psychosocial comorbidity.

## Abbreviations

ACHD: Adults with congenital heart disease
ASO: Arterial switch operation
CHD: Congenital heart disease
TGA: Transposition of the great arteries
HRQOL: Health-related quality of life
NYHA: New York heart association
QOL: Quality of life
